# Iterative Patient Testing of a Stimuli-Responsive Swallowing Activity Sensor to Promote Extended User Engagement During the First Year After Radiation: Multiphase Remote and In-Person Observational Cohort Study

**DOI:** 10.2196/47359

**Published:** 2024-02-28

**Authors:** Eileen H Shinn, Adam S Garden, Susan K Peterson, Dylan J Leupi, Minxing Chen, Rachel Blau, Laura Becerra, Tarek Rafeedi, Julian Ramirez, Daniel Rodriquez, Finley VanFossen, Sydney Zehner, Patrick P Mercier, Joseph Wang, Kate Hutcheson, Ehab Hanna, Darren J Lipomi

**Affiliations:** 1 Department of Behavioral Science University of Texas MD Anderson Cancer Center Houston, TX United States; 2 Department of Radiation Oncology University of Texas MD Anderson Cancer Center Houston, TX United States; 3 Department of Chemistry and Biochemistry College of Science University of Notre Dame South Bend, IN United States; 4 Department of Biostatistics University of Texas MD Anderson Cancer Center Houston, TX United States; 5 Department of Nano and Chemical Engineering University of California San Diego, CA United States; 6 Department of Electrical and Computer Engineering University of California San Diego, CA United States; 7 Department of Head and Neck Surgery University of Texas MD Anderson Cancer Center Houston, TX United States

**Keywords:** user-centered design, patients with head and neck cancer, dysphagia throat sensor

## Abstract

**Background:**

Frequent sensor-assisted monitoring of changes in swallowing function may help improve detection of radiation-associated dysphagia before it becomes permanent. While our group has prototyped an epidermal strain/surface electromyography sensor that can detect minute changes in swallowing muscle movement, it is unknown whether patients with head and neck cancer would be willing to wear such a device at home after radiation for several months.

**Objective:**

We iteratively assessed patients’ design preferences and perceived barriers to long-term use of the prototype sensor.

**Methods:**

In study 1 (questionnaire only), survivors of pharyngeal cancer who were 3-5 years post treatment and part of a larger prospective study were asked their design preferences for a hypothetical throat sensor and rated their willingness to use the sensor at home during the first year after radiation. In studies 2 and 3 (iterative user testing), patients with and survivors of head and neck cancer attending visits at MD Anderson’s Head and Neck Cancer Center were recruited for two rounds of on-throat testing with prototype sensors while completing a series of swallowing tasks. Afterward, participants were asked about their willingness to use the sensor during the first year post radiation. In study 2, patients also rated the sensor’s ease of use and comfort, whereas in study 3, preferences were elicited regarding haptic feedback.

**Results:**

The majority of respondents in study 1 (116/138, 84%) were willing to wear the sensor 9 months after radiation, and participant willingness rates were similar in studies 2 (10/14, 71.4%) and 3 (12/14, 85.7%). The most prevalent reasons for participants’ unwillingness to wear the sensor were 9 months being excessive, unwanted increase in responsibility, and feeling self-conscious. Across all three studies, the sensor’s ability to detect developing dysphagia increased willingness the most compared to its appearance and ability to increase adherence to preventive speech pathology exercises. Direct haptic signaling was also rated highly, especially to indicate correct sensor placement and swallowing exercise performance.

**Conclusions:**

Patients and survivors were receptive to the idea of wearing a personalized risk sensor for an extended period during the first year after radiation, although this may have been limited to well-educated non-Hispanic participants. A significant minority of patients expressed concern with various aspects of the sensor’s burden and its appearance.

**Trial Registration:**

ClinicalTrials.gov NCT03010150; https://clinicaltrials.gov/study/NCT03010150

## Introduction

### Background

In 2021, approximately 32,000 Americans developed laryngeal or pharyngeal cancer, which has a 5-year survival rate of 61% for all stages combined [1]. Management of these cancers often includes high-dose intensity-modulated radiation therapy (IMRT) designed to spare pharyngeal muscles and reduce the incidence of radiation-associated dysphagia (swallowing difficulty) [2]. Still, a range of studies have reported that roughly 60% of patients receiving IMRT developed long-term swallowing problems within 2 years after radiation had ended, ranging in intensity from inability to swallow solid food without compensatory strategies to being completely feeding tube dependent [3-10].

As with most chronic conditions, early detection and intensive swallowing therapies are key to preventing long-term dysphagia [11-26], especially if patients are adherent to swallowing therapy instructions [27]. However, noninvasive screening procedures for early detection of radiation-associated fibrosis do not yet exist in the United States. Instead, gold standard modified barium swallow (MBS) and fiberoptic endoscopic evaluation of swallowing tests are typically ordered after the patient begins to complain of difficulties with swallowing [12]. Furthermore, preventive swallowing therapies are not always prescribed prior to the development of radiation-associated dysphagia [28-30]. Unfortunately, once radiation-associated dysphagia is clinically detected, there is little hope of fully restoring normal function [11,31,32].

To detect radiation-associated dysphagia before it becomes permanent, it is necessary to monitor changes in swallowing function much more frequently than is currently possible in the clinical setting. Subclinical change in swallowing activity or risk for dysphagia could be assessed during standard cancer surveillance visits, but increasing the periodicity of these visits would increase patient burden by requiring more frequent travel to the medical center for swallowing imaging and tests. Frequent at-home monitoring with wearable sensors between scheduled surveillance visits could address this gap in monitoring, especially if the sensors were designed to support decision-making regarding initiation of intensive speech language therapies [33]. To this end, researchers have developed myriad devices that can be worn on the skin and measure a range of mechanical, optical, biochemical, electrical, or acoustic signals with high fidelity [34-39].

However, sensor performance alone is not sufficient for improving health outcomes as patient engagement is also important [40]. Within the specific context of preventing dysphagia in survivors of head and neck cancer, repeated at-home monitoring over a period of months if not years is necessary to demonstrate a clinical advantage over current treatment paradigms. Unfortunately, most mobile technologies fail to engage patients over sustained durations, with most mobile health (mHealth) interventions for chronic disease reporting steep declines in use, some as high as 95% within the first few weeks, depending on the technology and context [41-43]. The most frequently cited reasons for discontinued use are decreased interest in the technology after its novelty abates, perceived lack of usefulness relative to burden, poor implementation of user experience, and frustration with technical issues [44-47].

To counter these barriers, it is widely agreed that user-centered testing be conducted in a sustained and iterative fashion during the design and development of new health technology. User-centered testing assesses the human technology interface by evaluating how well the technology incorporates into end users’ daily routines, habits, and capabilities, known loosely as user acceptability [40,48]. Beyond acceptability, technologies should be designed to maximize their potential to effect changes in patients’ attitudes and health behaviors. Oinas-Kukkonen and Harjumaa’s [49] persuasive system design model describes four categories of persuasive design principles that optimize the likelihood of health behavior change: task support (personalized design features that make it easier for users to achieve their goals), social support (leveraging interpersonal learning, eg, via online community forums), dialogue support (providing feedback to the user in a manner that helps the user move toward their goal, eg, with praise and rewards), and system credibility (the perceived clinical expertise embedded within the sensor output) [49]. Relatively few mHealth interventions conduct user-centered testing during technology development, which may be one reason for diminishing patient engagement and eventual abandonment [50-53]. In the US market, the user abandonment rate of fitness trackers is 50% within 6-12 months [44,54]. Patient abandonment rates are higher for those 70 years and older; one study found that 43% of their sample had abandoned their sleep and activity trackers within the first 2 weeks of use [55].

A recent review of 51 mHealth intervention studies targeting chronic diabetes, cardiovascular, or pulmonary diseases noted that diminished patient engagement was prevalent and posed a significant threat to effective use of the technology. Accordingly, nonsignificant effects on clinical markers outweighed significant findings two-to-one [42]. Therefore, our study explicitly addressed the design of a wearable sensor with the future intended use of home-based assessment for 9 months, starting with the third month after radiation to the 12th month. All design preferences and opinions were solicited within the context of sustaining engagement with the sensor for 9 months during the first year since repeated measurements over time would be needed to detect patterns of developing dysphagia in posttreatment patients.

### Goal of This Study

We assessed patient needs and preferred characteristics regarding the design of a wearable sensor to deliver personalized risk of dysphagia. Specifically, we assessed perceived barriers to wearing the sensor for 9 months, starting from the third month after the end of radiation treatment (to allow for healing from radiation skin burn) until the 12th month post treatment, and the impact of proposed design features on willingness to wear the sensor. In the first of three iterative user-centered tests, we surveyed a large cohort of survivors of head and neck cancer who were 4-5 years past radiation treatment to assess the perceived need for the sensor and desired design features for future prototypes. In study 2, we assessed user acceptability for a wired prototype sensor within a small sample of long-term survivors, oversampled for radiation-associated dysphagia. Finally, in the third user test, we tested a revised prototype on a sample of patients with head and neck cancer undergoing active treatment to get a better sense of competing priorities during a fraught time in their lives. The revised prototype included more elastic and comfortable materials for the strain sensor and custom-made dry electromyography (EMG) sensors, as opposed to commercial sensors. During the third test, we repeated our questions about user acceptability and willingness to wear the sensor for 9 months, as well as new questions about bidirectional feedback in the form of haptic (vibration) signaling.

## Methods

### Study 1

#### Design and Eligibility

Survivors of head and neck cancer who were still alive and who were already enrolled in a psychosocial parent study were asked to answer a questionnaire about a hypothetical throat sensor. Men and women were eligible for the parent study if they had received radiation with curative intent for oropharyngeal (stage II-IVb), laryngeal (II-IVb), hypopharyngeal (I-IVb), or nasopharyngeal cancer (I-IVb), or an unknown primary cancer with cervical metastases; were at least 2 years post treatment; were 18 years or older; and spoke English. Men and women were excluded if they had treatment for previous head and neck cancer; a history of previous head and neck surgery (previous biopsy, tonsillectomy, or tracheotomy were allowed); other cancer diagnoses, except nonmelanoma skin cancer; or a history of current oropharyngeal dysphagia unrelated to cancer diagnosis (eg, dysphagia due to underlying neurogenic disorder).

#### Recruitment and Data Collection Procedures

For the psychosocial parent study, all eligible patients were approached for recruitment at the radiation clinic’s radiation education class after being identified at the weekly multidisciplinary tumor board conference. The accrual rate for entry into the original parent study was 77%; demographic and disease information was collected at baseline. Those patients who were already enrolled in the psychosocial parent study and still alive (n=234) were contacted by phone to determine if they would answer optional questions about a hypothetical sensor to be worn on the throat. Patients who did not return calls after 5 attempts or did not have working phone numbers were not approached further for enrollment into study 1. After obtaining informed consent, participants completed the optional questionnaire administered either by REDCap, telephone, or mail at a single time point [56]. For mailed questionnaires, a research staff’s phone number was provided if the patient had any questions about the questionnaire.

#### Measures

**Figure 1:**
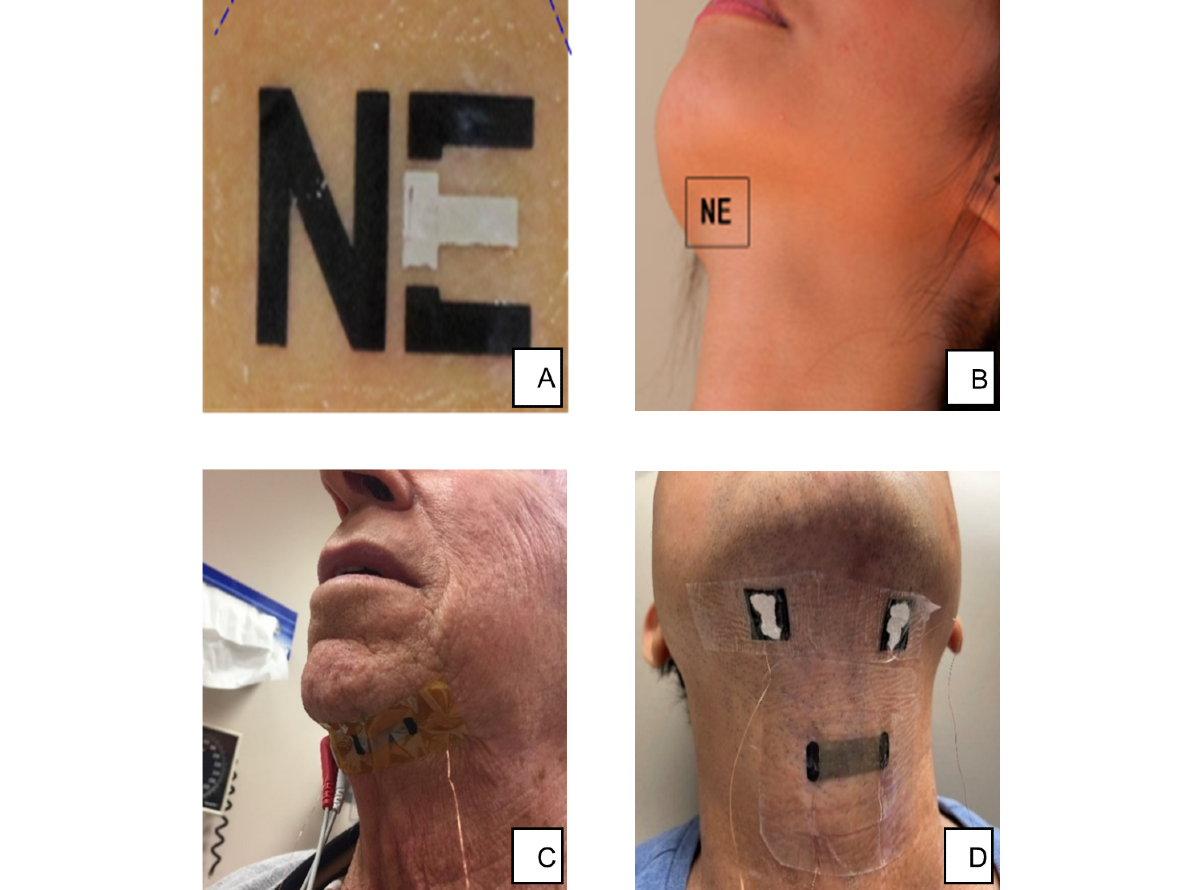
Appearance of the hypothetical and actual sensor prototypes. (A) Study 1 respondents were shown a photograph of the proposed sensor and (B) its proposed location on the neck. (C) Study 2’s graphene strain sensor prototype, supported on 13-µm-thick polyimide tape (the contact surface is silicone) and placed on the submental region probing muscle contraction. (D) Study 3’s soft polymer strain sensor, now placed under the laryngeal prominence to capture movement during swallowing.

Demographic information regarding age, race/ethnicity, employment, income, and marital status were obtained by questionnaire. Disease stage was abstracted from the medical record. Participants then completed a questionnaire. The first page of the questionnaire showed a photograph of the proposed sensor (Figure 1A) and a diagram of the sensor’s placement on the neck (Figure 1B), a brief description of the sensor’s purpose, and the proposed timeline of wearing the sensor every weekend from the third month post radiation to the 12th month post radiation for a total of 9 months.

#### Main Outcome: Willingness to Wear the Sensor

For studies 1-3, the study questionnaire asked whether the patient would have been willing to wear the sensor for 9 months during the first year after radiation, starting in month 3 post treatment. This time point was asked about since it would give sufficient time for the skin on their neck to have healed from radiation skin burn. Participants were then asked whether they would have been willing to wear the sensor for the entire 9 months, every other week, or every weekend during the 9-month period, and then a series of branched logic true-false questions about reasons for willingness versus unwillingness to wear the sensor. Next, using a 3-point Likert scale response format, all participants rated whether changes in the sensor design (either unobtrusive appearance or the ability to receive feedback about risk for dysphagia) would change the individual’s willingness or unwillingness to wear the sensor every weekend for 9 months. Additional comments or suggestions were also solicited as free text.

### Study 2

#### Design and Eligibility

A second sample of survivors of head and neck cancer who were 2 to 10 years post radiation and attending surveillance visits at MD Anderson gave informed consent and enrolled into the study during a 1-week period; testing was constrained to a 1-week period in which visiting graduate engineering students from the University of California San Diego traveled to MD Anderson for on-patient equipment testing. The eligibility criteria for study 2 were the same as for study 1; however, we oversampled for patients with a Dynamic Imaging Grade of Swallowing Toxicity score >0, indicating radiation-associated dysphagia that had been verified with MBS [57]. The oversampling was done to gauge the accuracy of the prototype sensor in distinguishing between dysphagic survivors and survivors without dysphagia [58]. For every dysphagic participant, we recruited a nondysphagic patient matched for age and sex. For patients who declined participation, deidentified disease information, demographics, and reason for refusal were noted in the study record.

#### Procedure and Assessment

A wired prototype graphene strain sensor coupled with a wired surface EMG sensor was placed on the patient to obtain muscle movement measurements during a series of swallowing tasks of various bolus textures, as described previously (Figure 1C) [58]. Immediately after the on-throat sensor test, patients were asked to answer six questions about the sensor’s discomfort, ease of use, and associated embarrassment using a 5-point Likert scale ranging from strongly disagree to strongly agree. Patients were again asked whether they would be willing to wear the sensor for 9 months (but now for once a month on the weekends) with branching questions asking for reasons for willingness versus unwillingness. Patients were again asked to rate the impact of sensor unobtrusiveness and predictive dysphagic feedback on willingness to wear the sensor for extended periods. Finally, demographic information regarding age, race, and marital status were abstracted from the medical record. All testing sessions were conducted at the Head and Neck Cancer Center at MD Anderson.

### Study 3

#### Design and Eligibility

Similar eligibility, consent, and testing procedures were used in study 3. However, eligible patients were more likely to be approached during active treatment for throat cancer, whereas studies 1 and 2 recruited long-term survivors. Study 3’s sensor (Figure 1D) was revised to have better skin conformation and comfort; standard surface EMG electrodes were now replaced with flexible custom dry electrodes, whereas the strain sensor was supported on a silicon substrate [59].

#### Assessment Procedures

After completion of the on-throat sensor test, patients were also asked the same questions used in study 1 regarding willingness to wear the sensor for 9 months and whether changes in the sensor’s appearance and feedback capability would change their minds about their willingness to wear the sensor. In addition, participants were interviewed regarding the helpfulness of future capability of the sensor itself to give immediate haptic feedback in three different scenarios: to indicate correct placement of the sensor, to indicate correct performance of a particular swallowing exercise, and to indicate quality of swallowing during at-home testing of various bolus textures. Their answers were transcribed, categorized, and coded into three categories (0: not helpful; 1: helpful under certain conditions; 2: helpful).

#### Analysis

Descriptive statistics (eg, proportions, means, ranges, and SDs) were computed for the process evaluation and participant satisfaction data, together with 95% CIs. To assess the external validity of the study, demographic and disease information was compared between respondents and nonrespondents in study 1 (Table 1) and between participants and refusers in studies 2 and 3 (data not shown; available data for participants in studies 2 and 3 is shown in Table 2). All questionnaire responses were analyzed with SPSS (version 26; IBM Corp).

**Table 1:** Demographic/disease comparisons between willing and unwilling participants (study 1).

Characteristic		Potentially eligible survivors (from parent study)					Survivors who completed the questionnaire							
		Total (N=234)	Nonrespondent (did not participate)	Respondent	*P* value		Total (n=138)		Willing to wear for 9 mo		Unwilling		*P* value	
**What is your age? (years)**						.28								.15
	Participants, n (%)	234	96 (41.0)	138 (58.9)			138		22 (15.9)		116 (84.1)			
	Mean (SD)	57.4 (10.0)	56.6 (9.8)	58 (10.1)			58 (10.1)		55.2 (9.4)		58.5 (10.1)			
	Median (min-max)	58 (18-83)	56 (30-79)	59 (18-83)			59 (18-83)		56.5 (35-75)		59 (18-83)			
**What is your ethnic background? n (%)**						.003								.59
	Hispanic or Latino	21 (9.1)	15 (16.0)	6 (4.4)			6 (4.4)		0 (0.0)		6 (5.2)			
	Not Hispanic or Latino	210 (90.9)	79 (84.0)	131 (95.6)			131 (95.6)		22 (100.0)		109 (94.8)			
**Race, n (%)**						.23								.53
	African American	10 (4.3)	6 (6.4)	4 (2.9)			4 (2.9)		1 (4.5)		3 (2.6)			
	American Indian or Alaska Native	1 (0.4)	1 (1.1)	0 (0.0)			0 (0.0)		0 (0.0)		0 (0.0)			
	Asian	6 (2.6)	1 (1.1)	5 (3.64)			5 (3.6)		1 (4.5)		4 (3.5)			
	Native Hawaiian or Pacific Islander	1 (0.4)	0 (0.0)	1 (0.7)			1 (0.7)		0 (0.0)		1 (0.9)			
	Non-Hispanic White	213 (92.2)	86 (91.5)	127 (92.7)			127 (92.7)		20 (90.9)		107 (93.0)			
**Education, n (%)**						.02								.26
	Some college and lower	112 (48.9)	54 (58.1)	58 (42.6)			58 (42.6)		7 (31.8)		51 (44.7)			
	Bachelor’s degree or higher	117 (51.1)	39 (41.9)	78 (57.4)			78 (57.4)		15 (68.2)		63 (55.3)			
**Employment status, n (%)**						.60								.18
	Full-time/part-time	145 (63.3)	57 (61.3)	88 (64.7)			88 (64.7)		17 (77.3)		71 (62.3)			
	Not employed	84 (36.7)	36 (38.7)	48 (35.3)			48 (35.3)		5 (22.7)		43 (37.7)			
**Marital status, n (%)**						.50								>.99
	Single living alone/married but living apart/separated/divorced/widow	46 (20.0)	21 (22.1)	25 (18.5)			25 (18.5)		4 (18.2)		21 (18.6)			
	Single but living with significant other/married living with spouse	184 (80.0)	74 (77.9)	110 (81.5)			110 (81.5)		18 (81.8)		92 (81.4)			
**Occupation, n (%)**						.07								.54
	Professional/managerial	143 (71.9)	51 (63.0)	92 (78.0)			92 (78.0)		16 (88.9)		76 (76.0)			
	Retail/service/labor	44 (22.1)	24 (29.6)	20 (16.9)			20 (16.9)		2 (11.1)		18 (18.0)			
	Student/unemployed	12 (6.0)	6 (7.4)	6 (5.1)			6 (5.1)		0 (0.0)		6 (6.0)			
**What is your income before taxes? (US $), n (%)**						.007								.22
	<30,000	38 (18.9)	24 (30.4)	14 (11.5)			14 (11.5)		0 (0.0)		14 (13.6)			
	30,000-50,000	31 (15.4)	13 (16.5)	18 (14.8)			18 (14.8)		2 (10.5)		16 (15.5)			
	50,000-75,000	28 (13.9)	9 (11.4)	19 (15.6)			19 (15.6)		2 (10.5)		17 (16.5)			
	>75,000	104 (51.7)	33 (41.8)	71 (58.2)			71 (58.2)		15 (78.9)		56 (54.4)			
**Stage of disease, n (%)**						.24								.70
	Stages I or II	76 (32.5)	27 (28.1)	49 (35.5)			49 (35.5)		7 (31.8)		42 (36.2)			
	Stages III or IV	158 (67.5)	69 (71.9)	89 (64.5)			89 (64.5)		15 (68.2)		74 (63.8)			

**Table 2:** Demographic/disease comparisons between willing and unwilling participants (studies 2 and 3).

Characteristic		Study 2						Study 3						
		Total sample (n=14)	Willing to wear for 9 mo (n=10)	Unwilling (n=4)	*P* value		Total sample (n=14)		Willing to wear for 9 mo (n=12)		Unwilling (n=2)		*P* value	
Age (years), mean (SD)		61.6 (11.5)	61.2 (12.3)	62.8 (11.0)	.83		62.4 (12.3)		61.0 (11.9)		70.5 (16.3)		.33	
**Race, n (%)**						.55								.57
	African American	0 (0)	0 (0)	0 (0)			0 (0)		0 (0)		0 (0)			
	American Indian or Alaskan Native	0 (0)	0 (0)	0 (0)			0 (0)		0 (0)		0 (0)			
	Asian	0 (0)	0 (0)	0 (0)			2 (14)		2 (17)		0 (0)			
	Native Hawaiian or Pacific Islander	0 (0)	0 (0)	0 (0)			0 (0)		0 (0)		0 (0)			
	Non-Hispanic White	13 (92.9)	9 (90)	4 (100)			12 (86)		10 (83)		2 (100)			
	More than one race	1 (7.1)	1 (10)	0 (0)			0 (0)		0 (0)		0 (0)			
**What is your ethnic background? n (%)**						.85								.70
	Hispanic or Latino	3 (21.4)	2 (20)	1 (25)			1 (7)		1 (8)		0 (0)			
	Not Hispanic or Latino	11 (78.6)	8 (80)	3 (75)			13 (93)		11 (92)		2 (100)			
**Occupation, n (%)**						.52								.87
	Managerial/professional	2 (14)	2 (20)	0 (0)			7 (50)		6 (50)		1 (50)			
	Retail, service, operator	9 (64)	6 (60)	3 (75)			6 (43)		5 (42)		1 (50)			
	Student or unemployed	3 (21)	2 (20)	1 (25)			1 (7)		1 (8)		0 (0)			
**Marital status, n (%)**						.37								.01
	Married/living with significant other	12 (86)	8 (80)	4 (100)			12 (86)		12 (100)		0 (0)			
	Single/divorced/widowed/separated	2 (14)	2 (20)	0 (0)			2 (14)		1 (50)		1 (50)			
**Dysphagic status, n (%)**		14			.27									
	Dysphagic (DIGEST^a^>0)	7 (50)	5 (50)	2 (50)	>.99		7 (50)		6 (50)		1 (50)		>.99	
	Not dysphagic (DIGEST=0)	7 (50)	5 (50)	2 (50)	N/A^b^		7 (50)		6 (50)		1 (50)		N/A	
**Disease stage, n (%)**						.14								.70
	I-II	1 (8)	0 (0)	1 (25)			1 (7)		1 (8)		0 (0)			
	III-IV	12 (92)	9 (90)	3 (75)			13 (93)		11 (92)		2 (100)			

^a^DIGEST: Dynamic Imaging Grade of Swallowing Toxicity.

^b^N/A: not applicable.

### Ethical Considerations

All study materials and procedures were approved by the institutional review board at MD Anderson Cancer Center’s institutional review board (protocol 2016-0597). All enrolled participants signed informed consent forms before testing began. All study data were deidentified, and no compensation was provided for participation.

## Results

### Overview

Prior to patient user testing, our study incorporated design input from multiple disciplines, including behavioral scientists, speech pathologists, radiation oncologists, and engineers. Initially, our primary concerns were to develop a wearable device that would not injure skin sensitized by radiation and have an uncomplicated application and removal procedure. Various invasive sensors, such as those worn inside the mouth, were dropped from consideration after it was realized that patients would possibly need to use the device during radiation and later at home during the first year post treatment. During study 1, we gathered patient reactions to a photograph of a sensor (Figure 1), whereas in studies 2 and 3, prototype versions were tested on survivors and patients in the clinic (Figure 1). The racial breakdown of the overall study sample (N=234) was non-Hispanic White (n=213, 92.2%), African American (n=10, 4.3%), Asian American (n=6, 2.6%), American Indian/Alaska Native (n=1, 0.4%), and Native Hawaiian/Pacific Islander (n=1, 0.4%).

### Study 1

**Figure 2:**
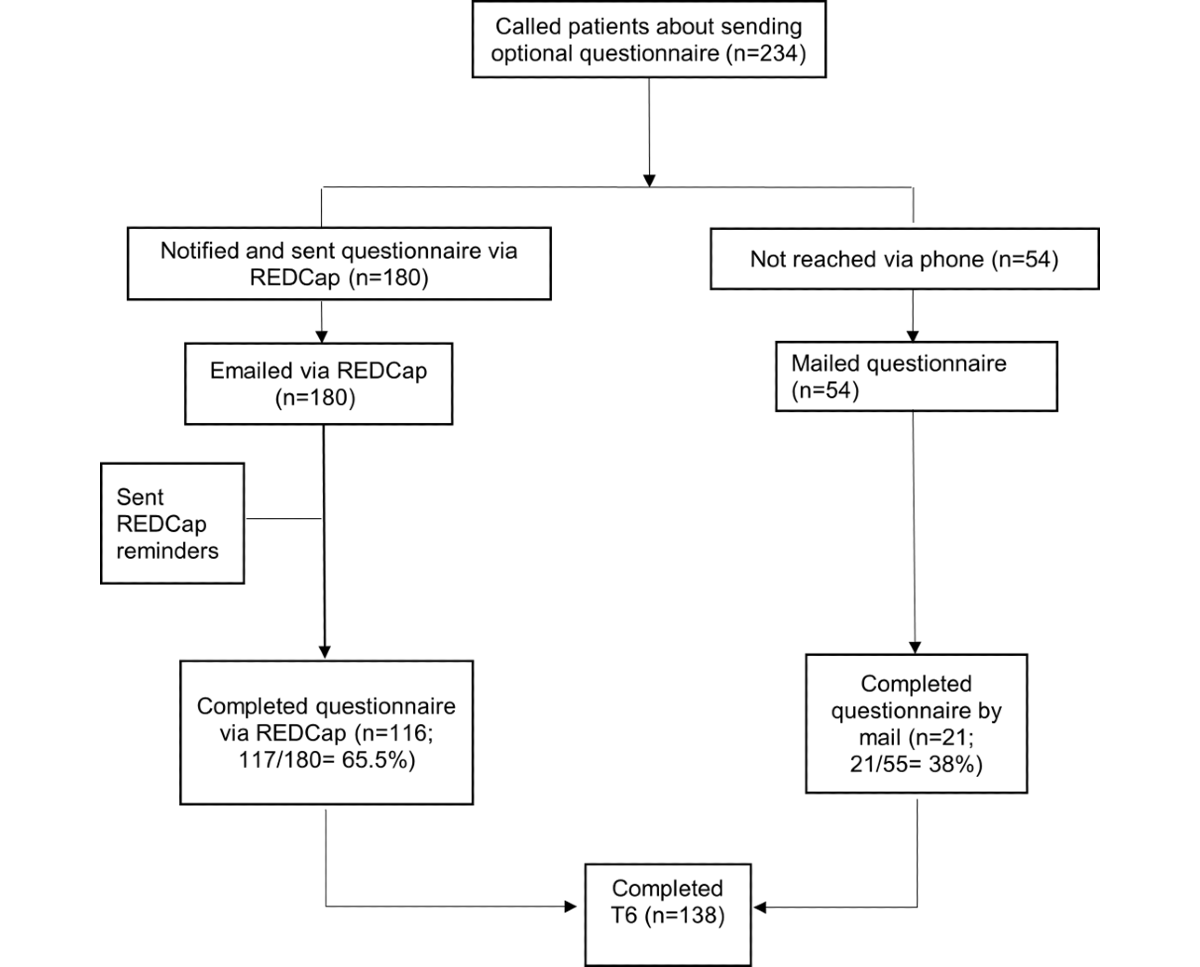
Recruitment CONSORT (Consolidated Standards of Reporting Trials) for study 1 (n=138).

Research staff contacted 234 eligible participants to complete study 1’s questionnaire, either via REDCap or by mail; 138 (59%) participants completed the questionnaire (Figure 2). Participants in study 1 were primarily non-Hispanic White and married, and their mean age was 57.4 (SD 10) years (Table 1). Median time since end of radiation treatment was 4 years and 26 days (Table 1). Analyses of responders versus nonresponders showed that responders were more likely to be non-Hispanic, have a bachelor’s degree, and have higher annual income; differences in race, age, and disease stage were not significantly different (Table 1).

### Survivor Preferences for Wearable Throat Sensor

Of the 138 respondents, 115 (83.3%) agreed that they would have been willing to wear the sensor for 9 months during the first year after radiation. However, patients were not willing to wear the sensor during the workweek due to fear of coworkers or strangers asking about the sensor. Instead, they were willing to wear the sensor on weekends, but only for one weekend a month as opposed to every weekend. When presented with several potential reasons explaining their willingness to wear the sensor, nearly all participants cited altruism, whereas 88% (92/105) cited interest in the sensor technology and 77% (75/97) thought that the sensor would help them adhere to their preventive swallowing exercises (Table 3). For example, several patients commented that the personalized feedback from the sensor would provide additional motivation to adhere to their preventive swallowing exercises:

It would push me to do my exercises diligently...

It would get me on the ball and do my exercises more often...

It would give me the information I can use to fight back the scar tissue problem. And see the importance of my neck exercises.

**Table 3:** Studies 1-3: Number of patients endorsing reasons for willingness/unwillingness to wear the sensor every weekend for 9 months.

Reasons for willingness/unwillingness to wear the sensor for 9 months^a^			Would wear sensor, n (%)					Would not wear sensor, n (%)		
			True		False		True			False
**Study 1 (n=138)^b^**										
	The technology of the patch sounds interesting.	92 (87.6)		13 (12.4)		—^c^			—	
	Wearing the patch would have reminded me to do my swallowing exercises.	75 (77.3)		22 (22.7)		—			—	
	I wanted to help with MD Anderson’s research.	108 (99.1)		1 (0.9)		—			—	
	My skin was still sensitive during that time.	—		—		11 (50)			11 (50)	
	I wouldn’t want to put on and take off the patch every weekend.	—		—		14 (63.6)			8 (36.4)	
	I wouldn’t want to wear the patch for 9 months.	—		—		19 (86.4)			3 (13.6)	
	I would feel uncomfortable if people noticed the patch and ask me questions or wanted to talk about it.	—		—		12 (57.1)			9 (42.9)	
	I was being asked to participate in too many studies.	—		—		1 (5.3)			18 (94.7)	
	It would have added to my daily responsibilities.	—		—		11 (55.0)			9 (45.0)	
	It would have been a reminder of my cancer treatment.	—		—		6 (30.0)			14 (70)	
	I would not be able to see my data from the patch.	—		—		6 (28.6)			14 (71.4)	
**Study 2 (n=14)^d^**										
	The technology of the patch sounds interesting.	8 (80)		2 (20)		—			—	
	Wearing the patch would have reminded me to do my swallowing exercises.	10 (100)		0 (0)		—			—	
	I wanted to help with MD Anderson’s research.	10 (100)		0 (0)		—			—	
	My skin was still sensitive during that time.	—		—		1 (25)			3 (75)	
	I wouldn’t want to put on and take off the patch every weekend.	—		—		2 (50)			2 (50)	
	I wouldn’t want to wear the patch for 9 months.	—		—		4 (100)			0 (0)	
	I would feel uncomfortable if people noticed the patch and ask me questions or wanted to talk about it.	—		—		2 (50)			2 (50)	
	I was being asked to participate in too many studies.	—		—		0 (0)			4 (100)	
	It would have added to my daily responsibilities.	—		—		3 (75)			1 (25)	
	It would have been a reminder of my cancer treatment.	—		—		1 (25)			3 (75)	
	I would not be able to see my data from the patch.	—		—		0 (0)			4 (100)	
**Study 3 (n=14)^e^**										
	The technology of the patch sounds interesting.	12 (100)		0 (0)		—			—	
	Wearing the patch would have reminded me to do my swallowing exercises.	12 (100)		0 (0)		—			—	
	I wanted to help with MD Anderson’s research.	12 (100)		0 (0)		—			—	
	My skin was still sensitive during that time.	—		—		0 (0)			2 (100)	
	I wouldn’t want to put on and take off the patch every weekend.	—		—		0 (0)			2 (100)	
	I wouldn’t want to wear the patch for 9 months.	—		—		1 (50)			1 (50)	
	I would feel uncomfortable if people noticed the patch and ask me questions or wanted to talk about it.	—		—		1 (50)			1 (50)	
	I was being asked to participate in too many studies.	—		—		0 (0)			2 (100)	
	It would have added to my daily responsibilities.	—		—		2 (100)			0 (0)	
	It would have been a reminder of my cancer treatment.	—		—		0 (0)			2 (100)	
	I would not be able to see my data from the patch.	—		—		0 (0)			2 (100)	

^a^Participants were asked the following question “Which of the following reasons would motivate you to wear the sensor every weekend for 9 months after radiation?”

^b^In study 1, 155 (83.5%) participants indicated that they would wear the sensor, while 23 (16.5%) participants indicated that they would not wear it.

^c^Not applicable.

^d^In study 2, 10 (71.4%) participants indicated that they would wear the sensor, while 4 (28.5%) participants indicated that they would not wear it.

^e^In study 3, 12 (85.7%) participants indicated that they would wear the sensor, while 2 (14.3%) participants indicated that they would not wear it.

Others valued the additional information that the sensor would provide:

I would be curious to know what is going on with my body...

I would have liked to have known what was happening to my throat...

It’s my neck! Why wouldn’t I want to know?

**Figure 3:**
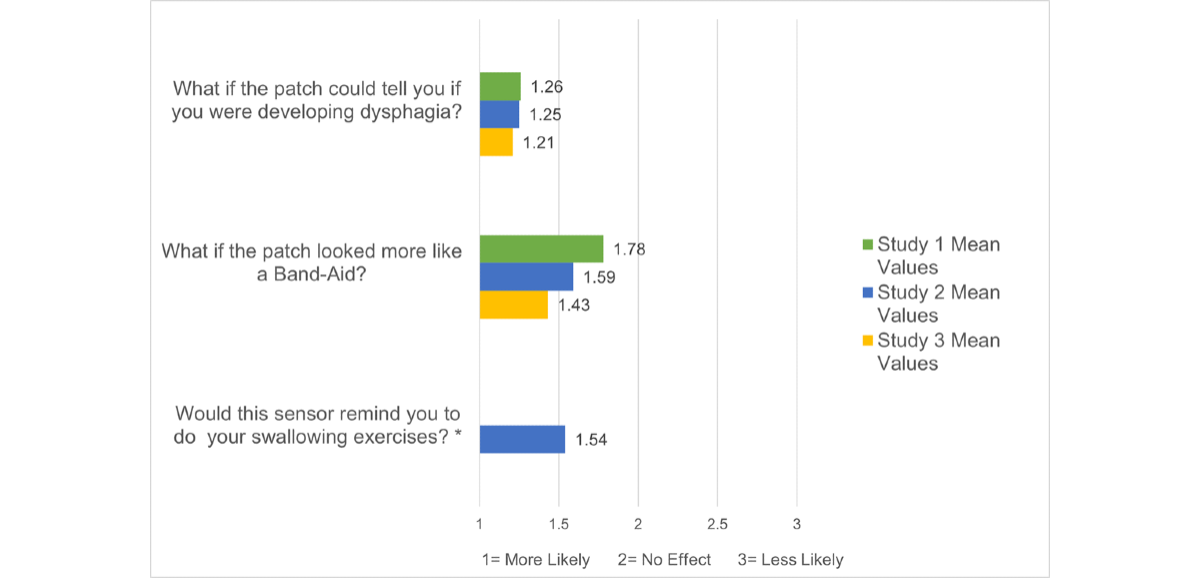
Studies 1 to 3: design feature impact on willingness to use the sensor for 9 months. *Only the participants in study 2 (n=14) were asked this question.

Among the 22 participants who indicated that they would have been unwilling to wear the sensor, nearly 90% (24/28, 85.7%) of all unwilling participants cited the lengthy duration of having to wear the sensor and 57% (16/28) disliked the idea of having to wear the sensor every weekend. The photograph of the proposed sensor had large black letters embedded within the sensor (Figure 1) to contain its wiring; over half of the unwilling participants objected to the sensor being noticeable enough that others would want to ask questions about its purpose. Just under one-third of unwilling participants disliked the idea of being reminded of their cancer treatment during the first year after radiation (Table 3). Participants who were unwilling to wear the sensor for 9 months did not have any significant demographic or clinical differences compared to participants who expressed willingness to wear the sensor. When asked whether changing the sensor’s appearance to that of a Band-Aid would impact willingness, 29% (4/14) of all study 1 participants agreed that this would increase their willingness, whereas 71% (10/14) stated that unobtrusive appearance would not affect their willingness (mean 2.45, SD 0.87; Figure 3):

Cosmetics is the least of my worries when I am going through treatment and fighting for my life.

When asked about the sensor’s proposed function of delivering individual risk for dysphagia, the majority of the sample (21/28, 75%) agreed that this feature would increase their willingness (mean 1.5, SD 0.88; Figure 3). Notably, half of the free-text comments indicated that had they been able to measure muscle fibrosis earlier, they would have been more diligent about performing their prescribed swallowing exercises. Some simply wrote that they wanted the sensor to be available so that future patients would understand that the risk of dysphagia was high:

I would like to see this in ACTION NOW

### Study 2

**Figure 4:**
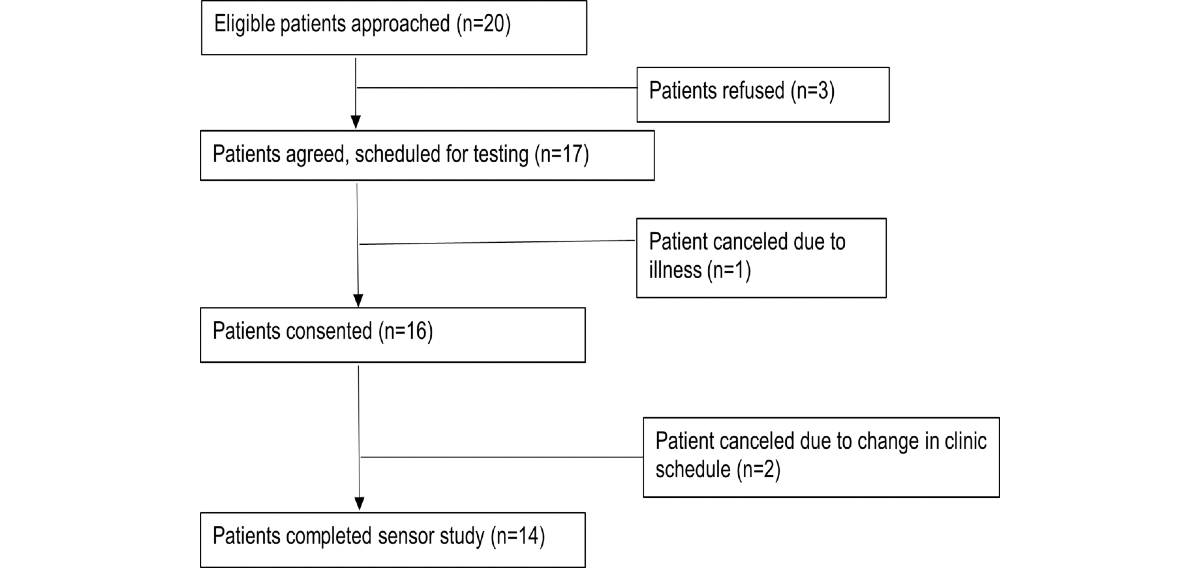
Recruitment flowchart for study 2 (n=14).

Within a 1-week period, a convenience sample of 20 potentially eligible survivors of oropharyngeal cancer who were nonmetastatic and able to speak English were approached at their surveillance visit for enrollment into the study. To test the sensor’s performance in distinguishing between normal and dysphagic swallowing patterns, survivors who had developed severe dysphagia as a result of their radiation were oversampled for study 2. Potentially eligible survivors were first identified in the electronic medical record; approached during a surveillance visit; and if consented, scheduled with the engineers for the sensor testing session in a clinic exam room. Three patients refused to participate, citing fatigue or disinterest; all were White, 2 were male, and 1 was female, and their age ranged from 63 to 74 years. Two of the patients were dysphagic and the third was nondysphagic. All three had been diagnosed with late-stage oropharynx cancer (data not shown). A total of 17 (85%) patients agreed, but 1 patient subsequently dropped out due to receiving news of cancer recurrence (Figure 4). Another 2 participants experienced scheduling conflicts; informed consent was obtained from the remaining 14 participants. Consistent with this cancer type’s demographic profile, the average age of the sample was 61 years, with 12 male participants and 2 female participants. Three participants were Hispanic or Latino and 3 were of non-White race (Table 2). Specific cancer diagnoses included cancer of the oropharynx (9/14, 64%), larynx (3/14, 21%), and nasopharynx (1/14, 7%), and unknown primary cancers (1/14, 7%). The average time since completion of radiation treatment was 47.9 months, and half of the sample had received a diagnosis of radiation-associated dysphagia (Table 2).

After wearing the sensor, 10 of the 14 (71%) patients indicated that they would have been willing to wear the sensor for 9 months of the first year post radiation. The most prevalent reasons for willingness were wanting to help future patients detect developing dysphagia and wanting to help MD Anderson research (Table 3). Of the 4 (29%) patients who did not think they would have been willing to wear the sensor, the most popular reason for unwillingness was study burden, specifically, that 9 months was too long of a testing period and the increased responsibilities associated with the sensor. Using a 5-point Likert response scale, patient ratings of discomfort (mean 1.21, SD 0.42), embarrassment (mean 1.14, SD 0.36), and difficulty in application and removal (mean 1.5, SD 0.52) were minimal (Table 4). Therefore, these questions were not repeated in the next phase of user testing.

**Table 4:** Study 2’s mean patient ratings for sensor discomfort, embarrassment, and difficulty of application (n=14), and study 3’s mean patient ratings of helpfulness for haptic signaling (n=14).

		Patient ratings, mean (SD)	Range^a^
**Study 2 (n=14)**			
	The sensor was uncomfortable to wear.	1.21 (0.426)	1.0-5.0
	The sensor would be difficult for me to use at home.	1.5 (0.519)	1.0-5.0
	I thought the experiment was fun.	3.79 (0.893)	1.0-5.0
	The testing session was embarrassing.	1.14 (0.363)	1.0-5.0
	I am good about doing my swallowing exercises every day.	3.27 (1.51)	1.0-5.0
	I believe it is important for me to do as many of my swallowing exercises as possible.	4.46 (1.13)	1.0-5.0
**Study 3 (n=14)**			
	Would it help for the sensor itself to vibrate when you put it in the right spot on your throat?	1.85 (0.376)	0-2.0
	Do you think it would be helpful to have the sensor vibrate once you did your swallowing exercise correctly?	2.00 (0.000)	0-2.0
	Do you think that having the sensor process your swallowing data and give you feedback about the quality of your swallowing would help?	1.46 (0.877)	0-2.0

^a^For study 2, the scale ranged from 1 (strongly disagree) to 5 (strongly agree). For study 3, the scale ranged from 0 (no) to 2 (yes).

### Study 3

**Figure 5:**
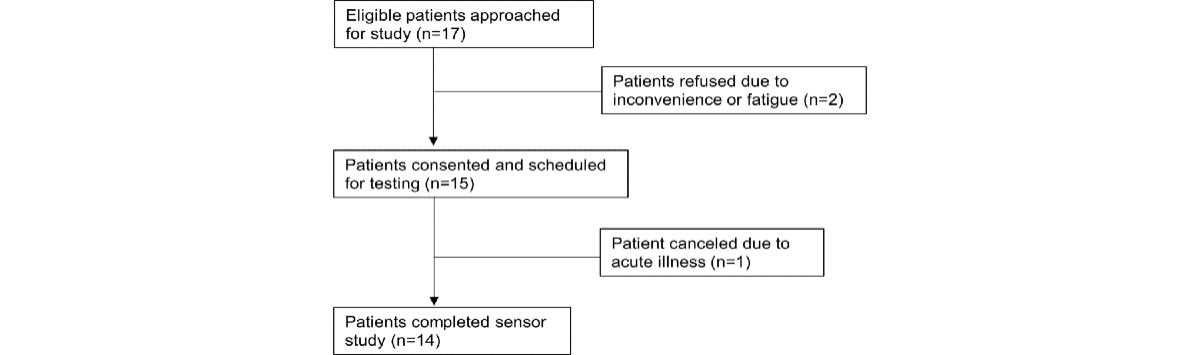
Recruitment flowchart for study 3 (n=14).

As with study 2, a convenience sample of 14 participants were recruited within a 1-week period to assess user preferences to the updated sensor prototype. As in the previous two studies, the majority of patients were diagnosed with oropharyngeal cancer (11/14, 79%). Unlike the previous two studies, 11 of the 14 (78.6%) were undergoing radiation at the time of testing; the remaining 2 participants were 1-5 year survivors (data not shown). The long-term dysphagic status was not yet known for patients on active treatment. A total of 17 participants were eligible and approached to participate in the sensor study. Two patients refused, both being White and male: 1 patient was aged 76 years and had been diagnosed with late-stage oropharyngeal cancer 2 years prior and the other was aged 23 years and was in the third week of radiation for late-stage oropharynx cancer (data not shown). A total of 15 (83%) patients agreed to participate and gave informed consent. One participant developed an acute illness episode the following day and was, therefore, unable to complete the sensor test, leaving 14 participants who completed user testing (Figure 5). Study 3’s sample was primarily male (12/14, 86%) and non-Hispanic White (12/14, 86%) with an average age of 62 years (Table 2).

As with the previous studies, the majority of patients (12/14, 86%) indicated willingness to wear the sensor for 9 months during the first year post radiation. Wanting to help future patients detect developing dysphagia and wanting to help MD Anderson research were the most prevalent reasons for willingness to wear the sensor (Table 3). As in study 2, the most oft-cited reasons for unwillingness were that of study burden (lengthy testing period and increase in daily responsibilities; Table 3). Patients’ opinions regarding the helpfulness of haptic feedback were obtained for 13 of the 14 participants. All 13 participants thought it would be helpful for the sensor to vibrate when placed in the correct spot on the neck (mean 1.85, SD 0.38) as well as when swallowing exercises were performed correctly (mean 2.0, SD 0.00; Table 4). A total of 11 (85%) participants felt it would be helpful for the sensor to give haptic feedback of swallow quality during at-home testing (mean 1.5, SD 0.88; Table 4).

## Discussion

### Principal Findings

To our knowledge, this is the first study to assess evaluations from patients with head and neck cancer of a wearable throat sensor in clinical settings with separate cohorts at varying time points along their treatment trajectory. Across all studies, the overall willingness to wear the sensor for 9 months during the first year after radiation was high and the perceived need was rated highly. However, study 1’s results should be interpreted with caution since the participation rate was 59%, with non-Hispanic and higher income/education patients more likely to complete the questionnaire. While study 2 and 3 used convenience samples for user testing, accrual rates were high (88%), even for those undergoing active treatment at the time of approach.

Direct comparison of our results with other works is not possible since the vast majority of published data regarding wearable devices equipped with mechanical, optical, biochemical, electrical, or acoustic sensors are pilot studies conducted with graduate students in a laboratory under highly controlled conditions [60-64]. While it did not test actual user engagement over repeated time points, it did gather patients’ opinions about the likelihood that they would wear the sensor for a period of several months. This question was asked in study 1 for patients who were only exposed to a photo of the proposed sensor, whereas patients and survivors in study 2 were asked this question after wearing the actual sensor while swallowing boluses of varying textures in a controlled setting. When searching for comparable studies that address extended user engagement with health technologies, the extant literature is limited to nonsensor research with mobile websites or apps [65] and to real-world studies of fitness tracker abandonment rates in healthy adults; these studies tend to describe a steep decline in user engagement over time. It is possible that our high rates of expressed willingness to wear the sensor for 9 months is due to the perceived usefulness of this device for this highly specialized problem.

Since the majority of participants (137/166, 83%) expressed willingness to wear the sensor for 9 months, data from those participants who were unwilling provided valuable insight into the potential barriers to its long-term use. Across all three studies, nearly 86% (24/28) of the unwilling participants perceived the 9-month testing period as too long. The second-most prevalent reason, that the sensor’s appearance would provoke unwanted attention, was endorsed by 56% (15/27) of the unwilling participants. The third-most frequent reason was an unwanted increase in daily responsibilities (16/26, 62%). This was also borne out by spontaneous comments in study 3, when nearly all 14 patients communicated a preference for a more streamlined one-step application process, rather than the separate applications for the strain sensor and surface EMG electrodes. On the other hand, several of the unwilling participants were much more willing to wear the sensor for 9 months if the sensor could provide individual dysphagic risk feedback and were made more unobtrusive in appearance (Figure 3). These findings are consistent with other mHealth reports citing multiple aspects of participant burden [48] and social implications of the technology’s appearance [66] as being relevant constructs to user engagement.

### Bidirectional Communication

Our data confirmed two other persuasive design principles: the desire for bidirectional communication (dialog support) with a team of clinical experts (system credibility). In all three studies, a large proportion of patients endorsed the rationale for the sensor (study 1: 115/138, 83.5%; study 2: 10/14, 71.4%; study 3: 12/14, 85.7%; ie, that sensor data be processed and sent back with contextual explanations of their risk of dysphagia development). Furthermore, of the three proposed persuasive design features, feedback about dysphagia risk had the greatest impact in increasing willingness among all participants (Table 4). These findings point to the importance of fostering a sense of connectedness and reassurance between the user and the technology so that patients’ association between their own health behaviors and subsequent health outcomes can be continually reinforced [42]. Future plans for implementation include data visualization of near-time individualized risk for dysphagia in the form of an app that can be linked with the throat sensor. When asked about direct haptic communication with the sensor itself, patients in study 3 rated haptics as helpful, especially when unsure about correct placement on the throat and whether preventive exercises were being done correctly (Table 4). One patient commented that he was never really sure if he was performing the exercises correctly at home and was “just winging it.”

### Sensor and Adherence to Exercises

The majority of participants (97/119, 82%) agreed that the sensor would serve as a reminder for them to do their speech pathology swallowing exercises. While the main goal of the sensor is to provide earlier detection of radiation-associated dysphagia, reminding patients to complete their swallowing exercises at home to counteract the development of dysphagia could be an additional benefit to this developing technology. Since personalized risk information is generally not sufficient in itself to increase exercise adherence per se [67], further user-centered testing would be needed to assess preferred modes of sensor feedback (eg, within an app or coupled with virtual coaching) [68].

### Limitations

Our study was conducted solely with survivors and patients attending clinical visits at MD Anderson, which generally requires high-quality insurance for access. Generalizability of our results are further limited by examining the demographic patterns among respondents versus nonrespondents in study 1. A total of 38% (21/55) of the eligible survivors did not complete the questionnaire despite repeated contact by the study team; nonresponders were significantly more likely to be Hispanic (*P*=.003), without a bachelor’s degree (*P*=.02), and of lower annual household income compared to respondents (*P*=.007). This is consistent with Rising et al’s [69] recent analysis of National Cancer Institute’s 2018 Health Information National Trends (HINTS) population survey data showing that nonusers of personal mHealth technologies were more likely to be older than 65 years and have lower incomes. Given the challenge of sustaining patient engagement in mHealth technology, future research should target these patients who fit within the above demographic profiles. Finally, the sample sizes for study 2’s and 3’s on-patient testing were constrained by the need to complete all testing within 1-week periods, as the sensors were applied/tested by visiting engineers and not MD Anderson research staff. It is conceivable that larger sample sizes might have produced a wider variation in response to the sensor’s features and perceived usefulness.

### Conclusion

Large proportions of non-Hispanic well-educated patients with high-quality insurance and above-average incomes were receptive to the idea of wearing a personalized risk sensor for an extended period during the first year after radiation. User ratings of discomfort and difficulty were minimal; however, a significant minority of patients expressed concern with various aspects of the sensor’s burden and its appearance.

## Abbreviations

EMG: electromyography
HINTS: Health Information National Trends
IMRT: intensity-modulated radiation therapy
MBS: modified barium swallow
mHealth: mobile health

## Data Availability

The data that support the findings of this study are available from the corresponding author EHS upon reasonable request.
